# The Research Progress in Targeted Therapy for Hypertension via Heat Shock Proteins

**DOI:** 10.3390/ijms27125586

**Published:** 2026-06-20

**Authors:** Bowen Sun, Yiming Jiao, Lin Lin, Xinhai Cui, Chao Li, Yunlun Li

**Affiliations:** 1 College of Traditional Chinese Medicine, Shandong University of Traditional Chinese Medicine, Jinan 250355, China; 2024100013@sdutcm.edu.cn (B.S.); jiaoyiming0415@163.com (Y.J.); cui_xinhai@126.com (X.C.); 2 Innovative Institute of Chinese Medicine and Pharmacy, Shandong University of Traditional Chinese Medicine, Jinan 250355, China; llinlin1221@163.com; 3 First Clinical Medical College, Shandong University of Traditional Chinese Medicine, 4655 University Road, Jinan 250355, China

**Keywords:** heat shock proteins, hypertension, mechanism, targeted therapy

## Abstract

As the core molecular chaperones of the cellular stress response, the heat shock protein (HSP) family has gained extensive attention for its role in the occurrence, development, and target organ damage of hypertension. This review aimed to comprehensively summarize the research progress of the HSP family in the field of hypertension, and to analyze its key roles in the pathogenesis of hypertension, including its regulatory effects on key pathological processes such as endothelial dysfunction, proliferation and migration of vascular smooth muscle cells, oxidative stress, and inflammatory responses. It also summarized the potential value of HSPs as biomarkers in the early diagnosis, condition monitoring, and prognostic evaluation of hypertension. Moreover, it discussed in depth the efficacy and safety of intervention strategies targeting HSPs, including the regulation of HSPs by gene editing, the targeted effects of small-molecule inhibitors, and the modulatory effects of natural products. We need to strengthen interdisciplinary collaboration mechanisms, accelerate the transformation of basic research results into clinical applications, carry out large-scale clinical trials, and develop specific modulators in the future, so as to ultimately provide solid scientific theoretical support and a practical clinical basis for the precise prevention and treatment of hypertension. The findings of this review not only provide novel insights into the pathogenesis of hypertension but also lay a theoretical foundation for the development of HSP-based biomarkers and targeted therapeutic strategies.

## 1. Introduction

Hypertension is a major driver of increased mortality and morbidity due to cardiovascular diseases (CVDs) [1]. Driven by increasing life expectancy and the growing prevalence of risk factors, hypertension and its adverse health impacts have been continuously rising [2]. By 2024, the number of patients with hypertension, aged 30–79 years, worldwide had reached 1.4 billion, but only approximately one-fifth of these patients attained effective disease control [3].

Heat shock proteins (HSPs) are a highly conserved protein family widely distributed in both prokaryotes and eukaryotes, whose core function is to preserve the structural and functional integrity of the cellular proteome [4]. HSPs are primarily classified into six major families based on their molecular weight and structural characteristics: large HSPs, HSP90, HSP70, HSP60, HSP40, and small HSPs. Besides acting as “molecular chaperones” in stress protection, HSPs can regulate the cell cycle, apoptosis, autophagy, and immune responses, thus exhibiting crucial biological significance in pathological conditions such as tumors, CVDs, and neurodegenerative disorders. HSPs have been demonstrated to be key regulators in the pathogenesis of hypertension. Accumulating evidence shows that specific HSP subtypes are closely involved in the core pathological processes underlying hypertensive vascular injury: endothelial dysfunction [5], abnormal proliferation and migration of vascular smooth muscle cells (VSMCs) [6], oxidative stress [7], and inflammation [8]. The functional consequences of HSPs in these processes are context-dependent and often paradoxical. For instance, HSP70 protects against angiotensin II-induced VSMC hypertrophy by inactivating ERK1/2 [9]. However, given its aberrant renal expression, it can also serve as an autoantigen to activate CD4+ T cells, thereby driving salt-sensitive hypertension [10]. Such duality positions HSPs not only as promising biomarkers for early diagnosis and prognosis but also as challenging, yet attractive, therapeutic targets. Existing reviews mostly focus on the basic functions of HSPs in CVDs, yet they lack subtype-specific and pathological link-specific elaboration for hypertension. Moreover, the dual role of HSPs in hypertension and their clinical translation potential as biomarkers and therapeutic targets remain to be comprehensively summarized.

The objective of this review was to comprehensively summarize the associations between HSPs and the pathological processes of hypertension based on recent experimental and clinical findings, thereby providing a scientific basis for HSP-based precision treatment of hypertension.

## 2. Methods

This narrative review summarizes recent advances in HSP research in the field of hypertension. Literature searches were conducted in PubMed and Web of Science from database inception through April 2026. The search terms were heat shock proteins, HSPs, hypertension, endothelial dysfunction, abnormal proliferation and migration of VSMCs, oxidative stress, inflammation, biomarker, targeted therapy, and their corresponding synonyms. The search was conducted by combining (using Boolean operators) the following search terms: “endothelial dysfunction * or abnormal proliferation and migration of vascular smooth muscle cells * or oxidative stress * or inflammation * or biomarker * or targeted therapy *” + “HSPs* or heat shock proteins *” and further refining by adding: “Hypertension”.

We prioritized original research articles, clinical studies, and authoritative reviews published in English. Key findings were narratively synthesized to clarify the regulatory mechanisms, biomarker potential, and therapeutic implications of HSPs in hypertension.

## 3. Regulatory Roles of HSPs in Key Pathological Processes of Hypertension

**Figure 1:**
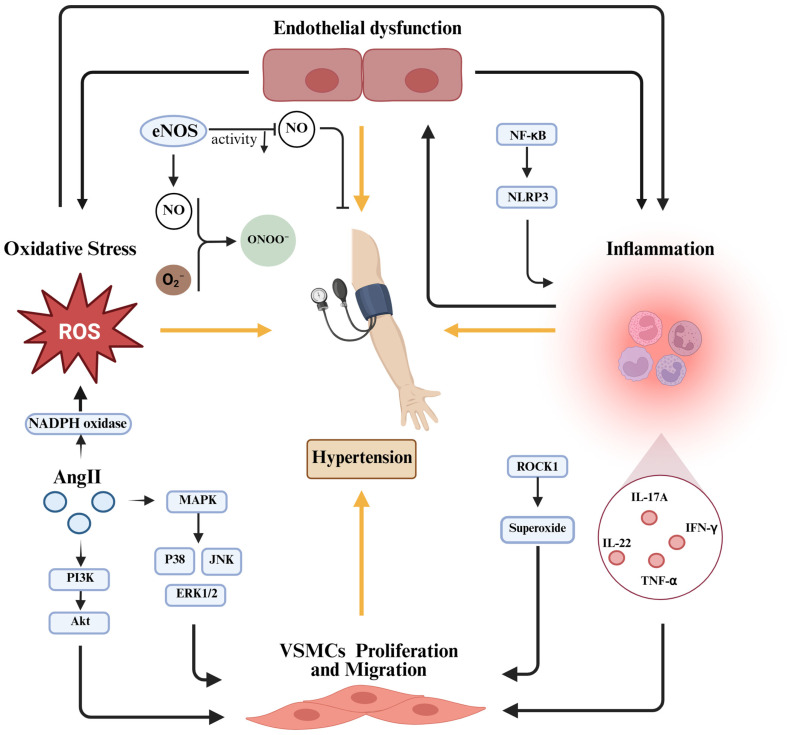
Interactions among key pathological processes in hypertension. Endothelial dysfunction, proliferation and migration of VSMCs, elevated oxidative stress, and activated inflammatory responses collectively constitute the core pathological cascade of hypertension. Endothelial dysfunction triggers oxidative stress and inflammation, resulting in reduced bioavailability of NO; decreased activity of eNOS reduces NO production. AngII activates NADPH oxidase, leading to excessive ROS generation. ROS not only depletes NO via ONOO^−^ formation but also promotes the release of canonical pro-inflammatory factors. IL-22 binds to endothelial cell receptors, thereby amplifying inflammatory responses and exacerbating endothelial dysfunction, all of which contribute to elevated blood pressure. AngII-mediated activation of inflammation and oxidative stress induces VSMCs proliferation. The MAPK, PI3K/AKT, and ROCK1 pathways are involved in regulating VSMCs proliferation and migration. The NF-κB pathway promotes the activation of the NLRP3 inflammasome, driving VSMCs proliferation, which leads to vascular remodeling and the progression and maintenance of hypertension. Created in https://BioRender.com. NO, Nitric Oxide; eNOS, endothelial Nitric Oxide Synthase; NF-κB, Nuclear Factor Kappa-B; NLRP3, NOD-like receptor pyrin domain-containing 3; ONOO^−^, Peroxynitrite Anion; O_2_^−^, superoxide anion; ROS, Reactive Oxygen Species; NADPH oxidase, Nicotinamide Adenine Dinucleotide Phosphate Oxidase; AngII, Angiotensin II; PI3K, Phosphatidylinositol 3-Kinase; Akt, Protein Kinase B; MAPK, Mitogen-Activated Protein Kinase; p38, p38 Mitogen-Activated Protein Kinase; JNK, c-Jun N-Terminal Kinase; ERK1/2, Extracellular Signal-Regulated Kinase 1/2; ROCK1, Rho-Associated Coiled-Coil Containing Protein Kinase 1; IL-17A, Interleukin-17A; IL-22, Interleukin-22; IFN-γ, Interferon-γ; TNF-α, Tumor Necrosis Factor-α.

Hypertension is a cardiovascular disorder characterized by persistent elevation of systemic arterial blood pressure, with intricate pathophysiological mechanisms centered on four core and interrelated processes: endothelial dysfunction, abnormal proliferation and migration of VSMCs, excessive oxidative stress, and aberrant inflammatory activation. These processes synergistically drive the progressive dysregulation of vascular structure and function, which is the core pathological basis of hypertension (Table 1 and Figure 1).

The vascular endothelium is the innermost layer lining the vascular wall, which plays a crucial role in regulating vascular tone, mediating inflammatory responses, and maintaining the homeostasis of the cardiovascular system [26]. Endothelial dysfunction is a key factor in the progression of hypertension [27]. In the development and progression of hypertension, endothelial dysfunction exerts its effects through hemodynamic abnormalities, overactivation of the Renin–Angiotensin–Aldosterone System (RAAS), and the resulting oxidative stress and inflammatory responses, collectively leading to reduced bioavailability of nitric oxide (NO), increased levels of vasoconstrictors such as endothelin-1 (ET-1), and ultimately a sustained elevation in peripheral vascular resistance and blood pressure [28,29]. Among these mechanisms, endothelial nitric oxide synthase (eNOS) is the key enzyme that synthesizes NO, a vasodilatory factor; reduced eNOS activity leads to diminished NO production, which in turn contributes to elevated blood pressure [30,31]. Moreover, NO not only dilates vascular smooth muscle and reduces vascular tone but also inhibits the proliferation of VSMCs and platelet adhesion [32,33].

VSMCs are the primary cellular components of the vascular wall, and their sustained contraction is essential for maintaining vascular tone and regulating blood flow [34]. VSMCs are responsible for maintaining vascular tone under physiological conditions. Also, their dysfunction induced by angiotensin II (AngII) and other growth factors, which includes enhanced oxidative stress, inflammation, migration, proliferation, and hypertrophy, plays a crucial role in the pathogenesis of hypertension and vascular remodeling [35]. Vascular remodeling is both a pathological alteration induced by hypertension and a factor that perpetuates and exacerbates the condition [36]. AngII directly enhances oxidative stress, inflammatory responses, and proliferation of VSMCs, thereby contributing to hypertensive vascular pathology [37]. Multiple signaling pathways are involved in regulating the contractility, proliferation, and migration of VSMCs, such as the MAPK pathway, PI3K/Akt pathway, and ROCK1 pathway [38,39]. The concept of vascular remodeling in the context of hypertension was first proposed in 1989 [40]. The abnormal proliferation and rearrangement of VSMCs, in addition to excessive extracellular matrix deposition, represent the key driving forces of hypertension-induced vascular remodeling; these changes ultimately promote the pathological progression of hypertension by increasing vascular resistance [41].

The essence of oxidative stress is an imbalance between reactive oxygen species (ROS) and the body’s antioxidant defense system [42]. ROS play a key role in the onset and development of hypertension by mediating the proliferation and migration of VSMCs, the regulation of endothelial function, and the modification of the extracellular matrix [43,44]. Mechanical stress and AngII associated with hypertension can markedly activate NADPH oxidase in vascular wall cells, rendering it the primary source of ROS [43,45]. ROS include superoxide anion (O_2_^−^) and hydrogen peroxide (H_2_O_2_); of these, O_2_^−^ rapidly combines with NO to form peroxynitrite anion (ONOO^−^) [46,47]. Excess ROS can activate the immune system and induce the release of inflammatory cytokines such as IFN-γ, interleukin (IL)-17A, and TNF-α. These factors directly promote sodium retention and vasoconstriction, thereby elevating blood pressure and forming a vicious pathological cycle [48].

Inflammation is one of the key factors in the course of hypertension, and immune disorders and inflammatory responses play an important role in the onset and progression of hypertension, target organ damage, and cardiovascular risk [49]. Neutrophils, γδ T cells, and others are all involved in the inflammatory and immune responses during hypertension [50,51]. Inflammatory markers such as IL-6, C-reactive protein (CRP), and TNF-α are upregulated to varying degrees in patients with hypertension [52,53,54]. Beyond classical pro-inflammatory factors, emerging immune cell subsets and the cytokines they secrete also play crucial roles. Notably, CD4^+^ T cells comprise multiple subsets—Th1, Th2, Th17, Treg, Th22, and others—each with specific cytokine profiles and effector functions. As a novel subset of CD4^+^ T cells, Th22 cells are significantly activated in hypertension and secrete IL-22. By binding to endothelial cell receptors and activating the STAT3 signaling pathway, IL-22 further amplifies inflammatory responses, exacerbates endothelial dysfunction, and ultimately contributes to the elevation of blood pressure [55]. IL-17A levels are also increased in patients with hypertension; independent of blood pressure, it directly acts on VSMCs of arterioles, inducing their hypertrophy and phenotypic switching and triggering arteriolar structural remodeling [56]. The balance between pro-inflammatory (Th17) and anti-inflammatory (Treg) subsets is often skewed toward inflammation in hypertension [57]. Moreover, CD4^+^ T cells are not merely direct effectors; they can recruit other immune cells, including monocytes, macrophages, and granulocytes, thereby amplifying local vascular inflammation [58]. CD4^+^ T cells possess remarkable phenotypic plasticity, such as the transformation of Tregs into Th17-like cells under inflammatory conditions [59]. This plasticity explains the conflicting results observed in some studies regarding the role of CD4^+^ T cells in hypertension and represents an important, but understudied, regulatory node in the pathogenesis of hypertension.

Hypertension can also promote increased histone acetylation and activate the NF-κB signaling pathway, thereby facilitating the transcription, assembly, and activation of the NLRP3 inflammasome. The activated inflammasome drives phenotypic transformation and proliferation of VSMCs, ultimately leading to vascular remodeling and the maintenance and progression of hypertension [60].

### 3.1. HSPs Regulate Endothelial Dysfunction

**Figure 2:**
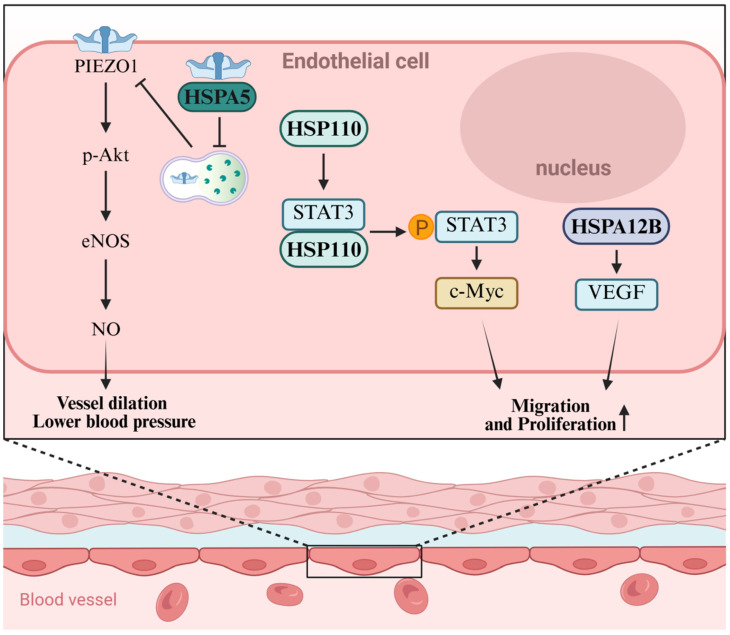
Role of HSPs in endothelial dysfunction. HSPs play a critical role in regulating vascular endothelial function. HSPA12B, a member of the HSP70 family, activates the VEGF signaling pathway and promotes proliferation and migration in HUVECs. Increased HSPA5 in endothelial cells stabilizes PIEZO1 by suppressing lysosomal degradation, activates the PIEZO1-Akt-eNOS pathway to promote NO production and alleviate AngII-induced hypertension. The interaction between HSP110 and STAT3 upregulates the expression of p-STAT3 and c-Myc, inducing abnormal proliferation and migration in HPAECs. Created in https://BioRender.com. PIEZO1, Piezo Type Mechanosensitive Ion Channel Component 1; HSPA5, Heat Shock 70 kDa Protein 5; HSP110, Heat Shock Protein 110; p-Akt, Phosphorylated Protein Kinase B; STAT3, Signal Transducer and Activator of Transcription 3; HSPA12B, Heat Shock 70 kDa Protein 12B; eNOS, endothelial Nitric Oxide Synthase; c-Myc, c-Myelocytomatosis Oncogene Protein; VEGF, Vascular Endothelial Growth Factor; NO, Nitric Oxide.

The HSP family bidirectionally modulates vascular endothelial functional homeostasis through multiple pathways. It plays a crucial role in the onset and progression of hypertension-associated endothelial dysfunction (Figure 2), and has potential as both an early biomarker of endothelial injury and a therapeutic target.

Clinical trials have demonstrated significantly higher serum HSP90α concentrations in patients with arterial hypertension than in normotensive controls, and their positive correlation with the severity of hypertension as reflected by systolic and diastolic blood pressure levels. These studies suggest that this elevation exerts a protective effect against early endothelial dysfunction in the pathogenesis of arterial hypertension, specifically, the enhanced HSP90α expression attempts to compensate for the impaired function of eNOS and the subsequent reduction in NO bioavailability [11].

Unlike the compensatory upregulation of HSP90α, dysregulated expression of HSP70 family members serves as a key regulatory driver of hypertension-associated endothelial injury. Among these, downregulated HSP70 expression directly impairs endothelial homeostatic maintenance. In vitro experiments using primary human endothelial cells showed that endothelial cells isolated from thrombus specimens of patients with chronic thromboembolic pulmonary hypertension undergoing pulmonary endarterectomy failed to effectively upregulate HSP70 expression under shear stress, ultimately driving impaired cell migration and microvascular dysfunction [16]. This directly confirmed that the normal expression of HSP70 was crucial for endothelial cells to respond to mechanical force stimulation and maintain their physiological functions. As key isoforms of the HSP70 family, HSPA12B and HSPA5 exert their biological functions through distinct signaling pathways. HSPA12B can promote the proliferation and migration of human umbilical vein endothelial cells by activating the vascular endothelial growth factor (VEGF) signaling pathway. In vitro studies have demonstrated that HSPA12B overexpression reverses the α-lipoic acid–induced inhibition of endothelial cell proliferation and migration [20]. In vivo studies in AngII-induced hypertensive mouse models have shown that increased expression of HSPA5 in endothelial cells stabilizes PIEZO1 by inhibiting its lysosomal degradation. This process activates the downstream PIEZO1-Akt-eNOS signaling axis and promotes the synthesis and release of NO, ultimately attenuating AngII-induced hypertension [5].

Not all members of the HSP family exert homeostatic effects on the vascular endothelium. Some isoforms can mediate endothelial dysfunction and pathological vascular remodeling and participate in the pathological progression of hypertension. Furthermore, in vitro and in vivo studies in pulmonary arterial hypertension models have demonstrated that HSP110 can drive the abnormal proliferation and migration of human pulmonary artery endothelial cells (HPAECs) through interacting with STAT3, thereby contributing to vascular remodeling [23]. Therefore, HSPs serve as both potential biomarkers of endothelial injury and therapeutic targets for improving vascular endothelial function; they maintain the homeostasis of vascular endothelial function by regulating endothelial cell proliferation and migration and the release of vasoactive substances through multiple pathways.

### 3.2. HSPs Regulate the Proliferation and Migration of VSMCs

**Figure 3:**
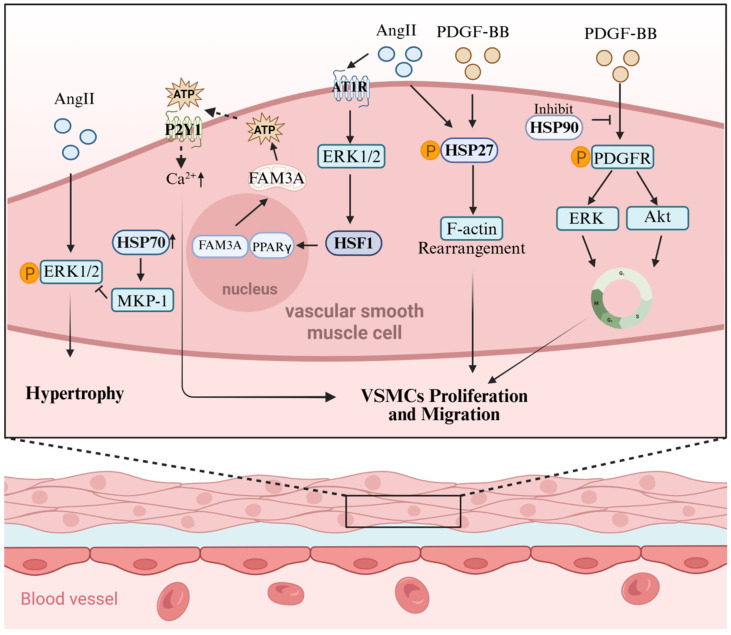
Role of HSPs in the proliferation and migration of VSMCs. AngII and PDGF-BB induce HSP27 phosphorylation, driving actin cytoskeleton rearrangement in VSMCs and enhancing cell migration. Meanwhile, HSP70 inhibits AngII-induced VSMCs hypertrophy by promoting MKP-1 accumulation and accelerating ERK1/2 inactivation. AngII drives abnormal proliferation and migration of VSMCs via the HSF1-FAM3A-ATP signaling axis. Inhibition of HSP90 expression induces G1-phase cell cycle arrest in VSMCs and suppresses cell proliferation. Created in https://BioRender.com. AngII, Angiotensin II; PDGF-BB, Platelet-Derived Growth Factor-BB; HSP27, Heat Shock Protein 27; HSP70, Heat Shock Protein 70; HSP90, Heat Shock Protein 90; ERK1/2, Extracellular Signal-Regulated Kinase 1/2; MKP-1, Mitogen-Activated Protein Kinase Phosphatase-1; FAM3A, Family With Sequence Similarity 3 Member A; ATP, Adenosine Triphosphate; P2Y1, Purinergic Receptor P2Y1; PPARγ, Peroxisome Proliferator-Activated Receptor γ; HSF1, Heat Shock Factor 1; PDGFR, Platelet-Derived Growth Factor Receptors; Akt, Protein Kinase B.

Members of the HSP family can directly modulate core pathophysiological behaviors of VSMCs, including proliferation, migration, hypertrophy, and cell cycle progression, which serve as the key initiating events of vascular remodeling in hypertension and atherosclerosis (Figure 3).

HSP70 plays a role in maintaining the homeostasis of VSMCs during vascular remodeling caused by hemodynamic changes [61]. Furthermore, in vitro cell culture experiments have demonstrated that HSP70 can significantly inhibit AngII-induced hypertrophy of VSMCs. These results show that HSP70 promotes MKP-1 accumulation, which accelerates ERK1/2 inactivation and thereby blocks the pro-hypertrophic signaling pathway [9].

Contrary to the stabilizing effect of HSP70, the small HSP27 is well established as a key mediator that maintains the proliferative and migratory capacities of VSMCs. In vitro studies have demonstrated that HSP27 expression directly modulates VSMC proliferation and markedly enhances VSMC migratory activity by regulating actin cytoskeleton rearrangement [62]. In vitro investigations have demonstrated that AngII and PDGF-BB induce the phosphorylation of HSP27, thereby driving the rearrangement of the actin cytoskeleton and enhancing the migratory capacity of VSMCs [22].

As the key upstream transcription factor governing the HSP family, HSF1 can also modulate VSMC function via a noncanonical metabolic signaling axis independent of the classical HSP transcriptional regulatory program. The HSF1-FAM3A-ATP signaling axis in VSMCs is a key regulatory pathway in AngII-induced hypertension. In vivo studies have confirmed that AngII activates HSF1 through the AT1R-ERK1/2 pathway. Activated HSF1 then transcriptionally upregulates FAM3A via PPARγ, and FAM3A further promotes ATP production and extracellular release in VSMCs. The released ATP increases intracellular Ca^2+^ levels through ATP-P2Y1 signaling and drives abnormal proliferation and migration of VSMCs, ultimately mediating the onset and progression of vascular remodeling and hypertension [6].

Contrary to the aforementioned small HSP, the high-molecular-weight molecular chaperone HSP90 displays pathological context-dependent expression profiles and functional features in VSMCs. Studies using in vitro and in vivo atherosclerotic models have shown that HSP90 is abnormally highly expressed in blood vessels with atherosclerotic plaque lesions; inhibition of HSP90 can induce G1-phase cell cycle arrest in VSMCs, accompanied by downregulation of cell cycle regulatory protein expression, thereby suppressing cell proliferation [12].

### 3.3. HSPs Regulate Oxidative Stress Responses

**Figure 4:**
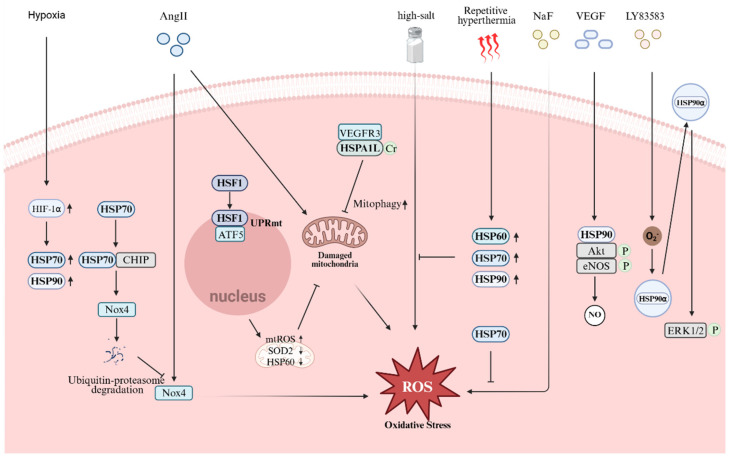
Role of HSPs in oxidative stress responses. The interaction between VEGFR3 and HSPA1L promotes crotonylation of HSPA1L, rescues AngII-induced mitochondrial autophagy defects, and alleviates oxidative stress injury. Upregulation of HSP70 suppresses NaF-induced oxidative stress; meanwhile, HSP70 acts synergistically with CHIP to promote ubiquitination and degradation of Nox4, thereby exerting an antioxidant effect. Induction of HSP60, HSP70 and HSP90 expression by repeated hyperthermia can inhibit high-salt-induced cardiac oxidative stress. Upon VEGF stimulation, HSP90 enhances phosphorylation of Akt and eNOS, which mediates NO release. Under oxidative stress conditions, LY83583-mediated generation of superoxide anion induces the secretion of HSP90α by VSMCs, which in turn activates the ERK1/2 signaling pathway. HIF-1α promotes the expression of HSP90 and HSP70 under hypoxia. Created in https://BioRender.com. NaF, Sodium Fluoride; VEGF, Vascular Endothelial Growth Factor; AngII, Angiotensin II; HSPA1L, Heat Shock 70 kDa Protein 1L; HSP70, Heat Shock Protein 70; HSP90, Heat Shock Protein 90; HSP60, Heat Shock Protein 60; HSF1, Heat Shock Factor 1; HIF-1α, Hypoxia-Inducible Factor-1α; CHIP, Carboxy Terminus of HSP70-Interacting Protein; Nox4, NADPH Oxidase 4; ATF5, Activating Transcription Factor 5; SOD2, Superoxide Dismutase 2; VEGFR3, Vascular Endothelial Growth Factor Receptor 3; ROS, Reactive Oxygen Species; Akt, Protein Kinase B; eNOS, endothelial Nitric Oxide Synthase; NO, Nitric Oxide; O_2_^−^, Superoxide Anion; ERK1/2, Extracellular Signal-Regulated Kinase 1/2; UPRmt, Mitochondrial Unfolded Protein Response; mtROS, Mitochondrial Reactive Oxygen Species.

Different isoforms of the HSP family display marked functional heterogeneity in the modulation of oxidative stress. These isoforms can participate in the pathological regulation of hypertension and its target organ damage through multiple mechanisms, including preserving mitochondrial functional homeostasis and regulating the expression of the endogenous antioxidant defense system (Figure 4).

In terms of renal protection against hypertensive injury, in vitro and in vivo studies have demonstrated that HSPA1L, a core member of the HSP70 family, can interact with VEGFR3 to regulate mitophagy, thereby reducing oxidative stress and blocking hypertension-induced renal injury [7]. Consistent with this protective effect, losartan upregulates HSP70 in renal tubular epithelial cells of spontaneously hypertensive rats (SHR), which then synergizes with CHIP to promote the ubiquitination of Nox4 and ultimately degrade it via the proteasomal pathway, thus exerting an antioxidant effect [17]. Accumulating exogenous intervention evidence further confirms the essential role of HSP70 in antioxidant defense. Oxidative stress induced by NaF is a core driving force of hypertension; quercetin exerts antioxidant, anti-inflammatory and anti-apoptotic effects via its multi-target mechanism, among which the upregulation of HSP70 expression is one of the key mechanisms [18]. Besides renal protection, HSP70 family members also exert extensive antioxidant effects on other target organ damage in hypertension. In SHR, elevated lipid peroxidation in the frontal cortex and hippocampus, together with neurotoxic stimulation, induces HSP72 expression, which makes HSP72 a pivotal biomarker of cellular injury and excitotoxicity [21]. In an experiment using Dahl salt-sensitive hypertensive rats, hypertension induced by a high-salt diet led to a significant increase in the levels of oxidative stress and inflammation in cardiac tissue; however, the induced expression of HSP60, HSP70, and HSP90 via repetitive heat therapy significantly inhibited this increase and preserved telomerase activity, implying the role of HSPs in preventing hypertension-related tissue injury [19].

In the pathological process of hypertensive cardiac remodeling, the activation of the mitochondrial unfolded protein response (UPRmt) pathway exerts a key antioxidant effect. In vitro and in vivo studies have demonstrated that by promoting the nuclear translocation of HSF1, it upregulates ATF5 in the UPRmt pathway, which further reduces mtROS and increases the expression of antioxidant enzyme superoxide dismutase 2 (SOD2) and HSP60. These effects inhibit oxidative stress damage and restore mitochondrial homeostasis, thus alleviating hypertension-induced cardiac remodeling and slowing the progression of hypertension [24].

Additional cellular experiments have demonstrated that upon VEGF stimulation, HSP90 facilitates the assembly of the protein complex composed of Akt and eNOS and mediates their serial activation cascade, leading to NO release [13]. Physiological levels of NO scavenge ROS and upregulate antioxidant systems, thereby inhibiting oxidative stress. The oxidative stress inducer LY83583 induces the generation of O_2_^−^ in VSMCs, which in turn triggers the secretion of HSP90α by these cells. Extracellular HSP90α activates the ERK1/2 signaling pathway [14]. These findings indicate that the protective effects of HSPs are constrained by a physiological expression threshold. Once their expression levels exceed the normal range, HSPs shift from stress-defensive factors to pathological drivers. Besides the aforementioned mechanical and chemical stimuli, hypoxia serves as a key physiological regulator of HSP expression. Most of the underlying molecular mechanisms have been elucidated in nonhypertensive models. However, hypoxia-inducible factor-1α (HIF-1α) can upregulate the expression of HSP90, HSP70, and other HSP genes under hypoxic conditions [63,64]. In the context of hypertension, hypoxic microenvironments frequently occur in the vascular wall, renal medulla, and other target organs, suggesting that this HIF-1α-HSP regulatory axis may also operate during hypertensive injury.

### 3.4. HSPs Participate in the Inflammatory Response

**Figure 5:**
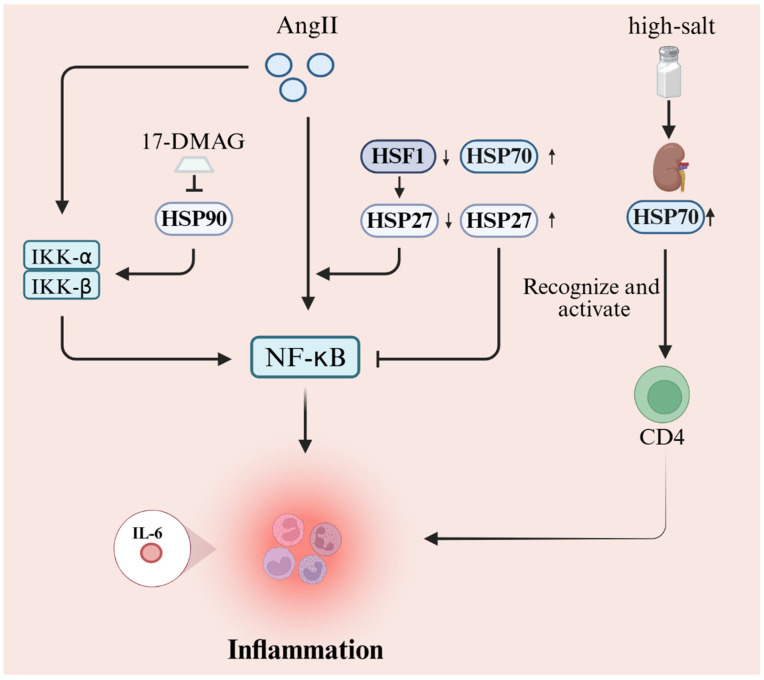
Role of HSPs in inflammatory responses. HSP70 plays a dual role in hypertension: under high-salt conditions, aberrantly expressed HSP70 in the kidney acts as an autoantigen to activate CD4+ T cells, triggering local renal inflammation. Upregulation of HSP70 acts synergistically with HSP27 to inhibit NF-κB activation and reduce IL-6 secretion, thereby protecting the vascular endothelium from AngII-mediated inflammatory injury. Knockdown of HSF1 in VSMCs downregulates downstream HSP27 and significantly exacerbates AngII-induced inflammatory responses. HSP90 mediates inflammatory responses by activating the NF-κB pathway, and inhibition of HSP90 activity can block AngII-induced inflammatory reactions. 17-DMAG blocks AngII-induced inflammatory signaling by downregulating IKK-α/IKK-β and suppressing NF-κB activation. Created in https://BioRender.com. AngII, Angiotensin II; 17-DMAG, 17-Dimethylaminoethylamino-17-Demethoxygeldanamycin; HSF1, Heat Shock Factor 1; HSP70, Heat Shock Protein 70; HSP90, Heat Shock Protein 90; HSP27, Heat Shock Protein 27; IKK-α, IκB Kinase α; IKK-β, IκB Kinase β; NF-κB, Nuclear Factor Kappa-B; IL-6, Interleukin-6.

HSPs are involved in the progression of inflammatory responses via dual mechanisms: on the one hand, they serve as endogenous antigens to activate the immune system; on the other hand, they can directly suppress pro-inflammatory signaling pathways (Figure 5).

Studies have shown that HSP70 plays a double-edged sword role in hypertension. It can activate immune cells via specific pattern recognition receptors to promote the expression of inflammation-related molecules [65]. In vivo studies in salt-sensitive hypertensive rat models have demonstrated that abnormally overexpressed renal HSP70 functions as a key autoantigen, which induces antigen-specific clonal expansion of CD4^+^ T cells. These activated CD4^+^ T cells secrete pro-inflammatory interleukin-6 (IL-6) to drive renal tubulointerstitial inflammation and impair pressure natriuresis, ultimately causing salt-sensitive hypertension [10].

Meanwhile, HSP70 also exerts an anti-inflammatory effect. In vivo investigations have found that the upregulation of HSP70 in conjunction with HSP27 can significantly suppress the activation of nuclear transcription factor NF-κB, thereby reducing the release of IL-6 and protecting vascular endothelium against AngII-mediated inflammatory damage [8]. This conclusion was further validated in studies on the upstream regulation of the HSF1-HSP27 signaling axis. In vitro loss-of-function studies have confirmed that the knockdown of HSF1 in VSMCs significantly downregulates the expression of its downstream HSP27, leading to a marked increase in the activation of AngII-induced pro-inflammatory transcription factor NF-κB [25]. Two independent studies, employing two orthogonal experimental dimensions of gain-of-function overexpression and loss-of-function knockdown, have further corroborated the core mechanism whereby HSP27 elicits its anti-inflammatory actions through suppressing the NF-κB signaling cascade.

Distinct from the bidirectional or predominant anti-inflammatory effects of HSP70 and HSP27, HSP90 primarily exerts pro-inflammatory effects in the modulation of inflammatory responses associated with hypertension. As a key regulatory protein, HSP90 activates inflammatory responses by triggering the NF-κB signaling pathway in multiple diseases [66,67]. In vivo studies in AngII-induced hypertensive mouse models have demonstrated that HSP90 inhibitor 17-DMAG can effectively block the inflammatory signaling pathway induced by AngII. It acts by downregulating the protein levels of IKK-α and IKK-β and inhibiting NF-κB activation, thereby reducing macrophage infiltration in the vascular wall and alleviating vascular inflammation [15]. This study not only reversely validated the pro-inflammatory mechanism of HSP90, but also provided key preclinical evidence for the application of HSP-targeted therapeutics in the prevention and treatment of hypertensive vascular injury.

The most striking contradiction in this field is the dual role of HSP70 in hypertension-related immunity. On one hand, HSP70 acts as an autoantigen that activates CD4^+^ T cells and promotes salt-sensitive hypertension; on the other hand, it suppresses NF-κB and reduces IL-6 secretion. Rather than dismissing this as an inconsistency, we suggest that this duality may be partially explained by at least two factors, although definitive evidence regarding this is still lacking. One possibility is that compartmentalization matters: stress-induced release of HSP70 into the kidney can trigger local autoimmunity, whereas the same protein retained inside the aortic wall or VSMCs can primarily limit cytokine production. Another contributing factor may be the chronicity and context of stress. Acute upregulation of HSP70 following heat shock or transient AngII infusion appears to be protective, but persistent overexpression under a high-salt diet can overwhelm degradation pathways and present neo-antigens.

HSPs serve as core bidirectional regulatory molecules throughout the entire pathological course of hypertension, encompassing both the onset and progression of hypertension as a unified whole and independent target organ damage. HSPs are comprehensively involved in four core pathological processes via multi-target and multi-pathway actions: vascular endothelial dysfunction, vascular remodeling mediated by aberrant proliferation and migration of VSMCs, oxidative stress injury, and chronic inflammatory activation. Also, they possess dual clinical value as both early biomarkers and targeted intervention targets for hypertension.

## 4. Potential Clinical Application Value of HSPs as Biomarkers for Hypertension

Given the crucial role of HSPs in the key pathological processes of hypertension, accumulating evidence has identified HSPs as potential biomarkers for the clinical management of hypertension, including early diagnosis, disease monitoring and prognostic evaluation (Table 2).

### 4.1. Research Progress of HSPs as Biomarkers for the Early Diagnosis of Hypertension

In the clinical management of hypertension, early identification and risk stratification are crucial for the prevention of target organ damage. Given their high sensitivity to vascular stress responses, HSPs have been increasingly recognized as a highly promising circulating biomarker in recent years.

The HSP70 family is the most extensively studied subtype of the HSPs to date. Animal experiments have demonstrated that renal HSP70 expression is significantly upregulated in two classic hypertensive models induced by AngII and L-NAME, respectively; furthermore, renal HSP72 expression is already upregulated in advance prior to the onset of persistent blood pressure elevation in SHRs [79,80]. Clinical studies have further validated the aforementioned findings: plasma HSP70 concentrations are significantly higher in patients with essential hypertension than in healthy controls, and its levels are significantly positively correlated with multiple inflammatory biomarkers including IL-6, TNF-α, and CRP, suggesting that HSP70 may act synergistically with pro-inflammatory cytokines to participate in the onset and progression of hypertension [68]. The early warning value of HSP70 is not limited to essential hypertension. Plasma HSP70 mRNA levels are significantly upregulated in patients with gestational hypertension, which can mirror the maternal pathological stress status [69]. The expression of HSP70 often precedes overt structural changes, making it a potential biomarker for predicting cardiovascular events [81]. Long-term follow-up studies have revealed that baseline serum HSP70 levels can predict the progression of atherosclerosis in patients with hypertension. The incidence of carotid intima-media thickness thickening during the 4-year follow-up period was lower in patients with high HSP70 levels, which may be attributed to the cytoprotective effects and immunomodulatory mechanisms of HSP70 [73].

HSP60 expression exhibits a certain degree of variability across various blood pressure stages. Clinical studies have revealed that serum HSP60 levels are significantly elevated in patients with borderline hypertension and positively correlated with carotid intima-media thickness [75]. These findings suggest that HSP60 may serve as a biomarker for early cardiovascular diseases and participate in the onset and progression of hypertension and atherosclerosis through its pro-inflammatory effects. However, subsequent studies have shown elevated levels of anti-HSP70 and anti-HSP65 antibodies in patients with established hypertension, and this elevation is independently associated with hypertension; however, no elevation in HSP60 levels has been observed [82]. This discrepancy in findings may be attributed to the persistent blood pressure fluctuations in patients with borderline hypertension, which continuously trigger stress responses and lead to elevated HSP60 levels in these patients. In contrast, the relatively stable blood pressure observed in patients with established hypertension does not elicit such a response.

Distinct from HSP70 and HSP60, the upregulation of HSP90α is recognized as a compensatory protective mechanism in response to endothelial injury induced by hypertension. Emerging research evidence indicates that hypertension-induced endothelial cell injury can induce increased secretion of HSP90α, which ameliorates endothelial function by stabilizing eNOS and promoting the production of NO [11]. Serum levels of HSP90α can indirectly reflect the severity of endothelial injury, yet its diagnostic cutoff value and clinical application value remain to be further validated in large-scale multicenter studies.

Despite promising associations, the clinical utility of HSPs as biomarkers for hypertension is still subject to substantial uncertainty. Discrepant findings for HSP60, which is elevated in borderline hypertension but not in established disease, suggest that HSP expression may vary with disease stage; however, cross-sectional studies cannot establish causality. One plausible interpretation is that HSP60 elevation represents an early, transient stress response to fluctuating hemodynamic forces rather than a sustained marker of established hypertension.

Taken together, although HSPs exhibit favorable diagnostic potential and HSP70, HSP60, and HSP90α display differential expression patterns in the early stages of hypertension, they still lack unified detection standards and diagnostic cutoff values as clinical biomarkers. Subsequent studies can further integrate multi-omics data to explore the combined application of HSPs with other cardiovascular biomarkers, so as to improve the specificity and accuracy of the early screening for hypertension and its complications.

### 4.2. Utilization of HSPs for Disease Monitoring of Hypertension: Feasibility and Efficacy

Hypertension, as a chronic progressive disease, has therapeutic goals not only involving controlling blood pressure values but also focusing on preventing the progression of target organ damage. Traditional blood pressure monitoring methods cannot fully reflect the degree of vascular injury and the status of target organ involvement. However, HSPs are core molecules in the body’s stress response, and the dynamic changes in their expression levels provide a novel perspective for the real-time monitoring of hypertensive conditions and exhibit remarkable clinical feasibility and efficacy.

In terms of monitoring disease progression, the expression levels of HSP family members are closely associated with the onset and progression of hypertension and can serve as potential indicators for tracking disease severity. Studies found that HSP27 exhibited age-related differences in expression levels in SHRs: its expression was significantly downregulated in 38-week-old rats and markedly upregulated in 57-week-old rats compared with the control rats [76]. This bidirectional change may represent a delayed adaptive protective response of the myocardium to long-term hemodynamic overload, which exerts myocardial protective effects by regulating the function of VSMCs. Similar to HSP27, HSP72, another key member of the HSP family, is also closely associated with hypertensive myocardial injury. Another study showed that the synthesis of myocardial HSP72 in SHRs was significantly enhanced in response to heat stress; however, despite the progressive aggravation of blood pressure and cardiac hypertrophy with age, the inducibility of myocardial HSP72 decreased markedly with aging [77]. This age-related decline in HSP72 inducibility may impair myocardial stress tolerance in elderly patients with hypertension and further increase the risk of cardiac remodeling, thus highlighting the value of HSP72 as a biomarker for assessing the aging-related progression of hypertensive myocardial injury. Beyond myocardial tissue, the expression of HSPs in vascular tissue also provides important clues for the monitoring of hypertensive vascular injury. Studies have demonstrated that acute blood pressure elevation by multiple vasoactive drugs can induce the expression of HSP70 in the rat aorta. Also, sodium nitroprusside prevents such drug-induced acute blood pressure elevation while simultaneously inhibiting HSP70 expression, suggesting a protective effect of HSP70 on blood vessels or maintain vascular homeostasis during hemodynamic stress [70]. The regulatory mechanisms of HSPs in hypertensive target organ damage also further support their monitoring value in the progression of hypertension. A previous study found that Asb10 synergistically drove pathological cardiac remodeling and cardiac function deterioration through pathways, including stabilizing HSP70, exacerbating inflammation, and promoting HDAC2 phosphorylation, in the TAC mouse model [71]. This finding not only clarifies the potential pathological role of HSP70 in late-stage hypertensive myocardial injury but also suggests that combined detection of HSP70 and its regulatory molecules may improve the accuracy of monitoring the severity of target organ damage.

Differences exist in the value of various members of the HSP family in hypertension monitoring. Subsequent prospective studies with larger sample sizes are needed to clarify the reference ranges, dynamic change thresholds and monitoring frequencies of different HSP indicators and further verify their applicability in different populations, so as to promote the standardized application of HSPs in the clinical monitoring of hypertension.

### 4.3. Potential Role of HSPs in the Prognostic Assessment of Hypertension

The prognostic assessment of hypertension is of high significance for the formulation of clinical intervention strategies and the prevention and control of adverse events. Recent studies have further confirmed that HSPs hold important potential value in the prognostic assessment of hypertensive patients, and they exhibit unique advantages, especially with respect to core prognostic indicators such as predicting the incidence of cardiovascular events and the progression of target organ damage.

HSPs are associated with the pathogenesis of hypertension, but their immune response status may also serve as a biomarker for assessing disease progression and intervention efficacy. Animal experiments have shown that the induction of immune tolerance to HSP70 can completely prevent the occurrence of salt-sensitive hypertension; on the contrary, artificial induction of renal HSP70 overexpression can lead to elevated blood pressure in animals pre-sensitized to HSP70 [10]. Therefore, monitoring the immune reactivity to HSP70 may provide a new prognostic tool for evaluating the disease activity of patients with hypertension and predicting their response to specific interventional measures. Besides the involvement of HSP70 in the dysregulation of immune homeostasis in hypertension, the aberrant expression of HSP70 is also closely associated with the onset and prognosis of common clinical complications of hypertension. A previous study found that the levels of serum HSP70 (sHSP70) in patients with recent onset atrial fibrillation (ROAF) were higher. Also, sHSP70 was an independent factor associated with the presence of atrial fibrillation; the levels of sHSP70 were even higher in patients with failed cardiac cardioversion and recurrence within 1 year after cardioversion. Thus, the study suggested that sHSP70 may constitute a prognostic tool for assessing the possibility of successful cardiac cardioversion and recurrence in patients with essential hypertension after the onset of symptomatic ROAF [72]. HSPs can also respond to external interventions and reflect the cardiovascular homeostasis and antioxidant capacity of patients with hypertension. Patients with hypertension with active physical activity and detectable plasma extracellular HSP72 (eHSP72) exhibited higher antioxidant enzyme activity and lower levels of lipid peroxidation products, indicating that eHSP72 levels can serve as a biomarker for measuring the amount of physical activity required to improve the antioxidant defense capacity and cardiovascular health of such patients [78].

Distinct from the HSP70 family, which is mainly involved in immune regulation and systemic stress responses, HSP90α preferentially mediates local cardiac structural remodeling in hypertension. In vivo studies have shown that the serum levels of HSP90α are elevated in patients with hypertension and positively correlated with left ventricular hypertrophy. The knockdown of HSP90α can alleviate pressure overload-induced cardiac hypertrophy, fibrosis, dysfunction, and the elevation of ROS levels in a mouse model of pressure overload established by TAC [74]. Therefore, HSP90α is not only a biomarker associated with adverse cardiac structural changes but also a key pathological factor driving the progression of the disease itself, further reinforcing the dual value of HSP90α as both a therapeutic target and a prognostic assessment biomarker.

In conclusion, the expression levels of various subtypes of the HSP family (especially HSP70 and HSP90α) are closely associated with blood pressure levels, degree of target organ damage and prognosis in patients with hypertension. Future studies can focus on the key values and dynamic change rules of different HSP subtypes for the prognostic assessment of hypertension, so as to further verify their application value in clinical practice.

### 4.4. Technical Methods and Standardization Issues for the Detection of HSP Biomarkers

The accuracy and degree of standardization of HSP detection methods are the critical prerequisites for the clinical translation of HSPs as hypertension-related biomarkers.

Immunological detection techniques based on the specific binding of antigens and antibodies are the most widely used traditional methods in the current clinical detection of HSPs, mainly including enzyme-linked immunosorbent assay (ELISA), Western blot, and chemiluminescent immunoassay. ELISA, by virtue of its advantages of simple operation, controllable cost, and high throughput, is widely used for the quantitative detection of HSPs in body fluid samples such as serum and plasma. For example, clinical studies have indicated that the levels of serum HSP90α and transferrin measured by ELISA may act as promising biomarkers to predict therapeutic responses to IL-17 inhibitors among patients with nonradiographic axial spondyloarthritis [83].

Electrochemical immunosensor technology based on nanomaterial modification has provided a new approach for the highly sensitive detection of HSPs in recent years. Studies have reported that an amperometric amplification biosensor based on an ITO chip modified with PS-AuNPs@Cys/Au enhances the loading efficiency of antibodies on electrodes through incubation with HSP70, which lowers the limit of detection of HSP70 to 25.7 pg/mL with a linear range of 0.1–1000 ng/mL. It also exhibits better performance than traditional ELISA in analyzing blood samples from healthy individuals [84], thus providing the possibility for the early detection of low-abundance HSPs in patients. Molecular biological detection techniques have displayed distinct advantages in the diagnosis of infectious diseases due to their high sensitivity and specificity. For the diagnosis of leishmaniasis, clinical studies have shown that the SYBR Green quantitative polymerase chain reaction assay targeting the HSP20 gene has a detection sensitivity of 88% and a specificity of 100%, and thus is significantly superior to the traditional microscopic detection method and is particularly suitable for rapid diagnosis in resource-scarce areas [85]. LAMP-Leish/HSP70, designed based on the HSP70 gene, also shows good accuracy in analyzing biopsy samples of cutaneous leishmaniasis, providing a new scheme for rapid clinical diagnosis [86].

At present, the standardization of HSP detection is faced with multiple challenges, such as insufficient standardization of detection procedures and the lack of a unified verification system for technical methods. In summary, the diversification of HSP detection technologies provides more options for various clinical scenarios, and the improvement in the standardization system is the key to breaking through the bottleneck in the clinical translation of HSPs. We need to establish a unified reference material system, formulate standardized detection procedures and verification specifications, and promote multicenter clinical studies to accumulate data, so as to fully exert the clinical value of HSPs as biomarkers in the future.

## 5. Targeted Intervention Strategies and Research Progress of HSPs in Hypertension

The clear causal link between HSP isoforms and the pathogenesis of hypertension, together with their biomarker value, further substantiates the feasibility of HSP-targeted intervention strategies for hypertension management. Several targeted therapies have been developed based on the roles of HSPs in hypertension and their potential as biomarkers. These include gene editing, small-molecule inhibitors, and natural products, especially active components from traditional Chinese medicine. These methods have displayed promising results in preclinical studies, and combining them helps overcome the limitations of using a single therapy alone (Table 3).

### 5.1. Prospects of Gene Editing–Based Targeted Regulation of HSPs in Hypertension Therapy

Genome editing technology refers to a series of technologies that can manipulate cellular DNA sequences by generating nuclease-mediated site-specific DNA breaks at desired genomic loci and resolving them through DNA repair pathways. Among these, the CRISPR-Cas system guides Cas nucleases to specific genomic loci via single-guide RNAs to achieve targeted manipulation of DNA sequences [95].

Traditional antihypertensive drugs mainly control blood pressure by blocking receptors or inhibiting enzyme activity. In contrast, the CRISPR/Cas9 technology offers the possibility of curing hypertension by precisely editing hypertension-related genes. Therefore, the development of gene editing technology is expected to break through the limitations of traditional intervention methods and become a new direction for the precision treatment of hypertension. As observed in animal models, the successful knockout or mutation of hypertension-associated genes using CRISPR/Cas9 has achieved a long-term and significant reduction in blood pressure [96,97], whereas the knockout of SNX1 led to increased expression of the AT1R protein in arteries, which in turn caused enhanced vasoconstriction and elevated blood pressure [98]. Studies in the field of tumor therapy have confirmed that CRISPR-Cas9-mediated knockout of the HSP90α gene can effectively inhibit the proliferation and metastasis of tumor cells, offering a novel strategy for precision cancer treatment [87]. Clinical evidence regarding gene editing strategies directly targeting HSPs for hypertensive management is lacking. Nonetheless, existing studies have confirmed the safety and efficacy of CRISPR-Cas9-based genetic intervention in the cardiovascular compartment and verified the viability of HSPs as druggable therapeutic targets. These findings establish a robust theoretical and technical basis for the future development of HSP-directed gene therapy in hypertension.

In general, gene editing technology provides an entirely new approach for the precision treatment of hypertension by targeting HSPs. With continuous iteration and optimization, this technology is expected to become a potential strategy for breaking through the limitations of traditional drug therapy and providing a potential long-term therapeutic strategy for hypertension.

### 5.2. Research Status and Challenges of Small-Molecule Inhibitors Targeting HSPs for the Treatment of Hypertensive Target Organ Damage

HSPs have emerged as potential therapeutic targets for treating cardiovascular complications. The core mechanism of action of small-molecule inhibitors targeting HSPs mainly revolves around interfering with the chaperone functions of HSPs, thereby blocking HSP-mediated abnormal signaling pathways.

HSP90 is one of the most abundant intracellular molecular chaperones, accounting for approximately 1–2% of total cellular proteins [99,100]. Studies have shown that, as HSP90 inhibitors, 17-AAG and 17-DMAG can inhibit key inflammatory signaling pathways and reduce the expression and secretion of inflammatory cytokines in cultured vascular cells. Also, 17-DMAG inhibits HSP90 and simultaneously blocks AngII-induced inflammatory response, extracellular matrix degradation and neovascularization [15]. Other studies indicate that 17-DMAG also ameliorates mitochondrial dysfunction, alleviates aortic adventitial fibrosis in AngII-induced hypertensive mice, and mildly reduces systolic blood pressure at the late stage [89]. In vitro and in vivo studies have found that 17-AAG attenuates cardiomyocyte-derived paracrine signaling to fibroblasts via HSP90 inhibition, thereby delaying the progression of myocardial interstitial fibrosis [88].

As the isoform with the largest molecular weight in the HSP family, direct studies on HSP110 in systemic hypertension remain relatively limited. Nevertheless, research advances in the field of PAH provide important insights for its application in hypertension. HSP110 can activate the downstream p-STAT3/c-Myc signaling pathway by forming a complex with STAT3, promote the abnormal proliferation and migration of pulmonary artery endothelial cells, and induce vascular remodeling; small-molecule inhibitors targeting the HSP110-STAT3 interaction can significantly ameliorate right ventricular hypertrophy and pulmonary vascular remodeling in rats with hypoxia-induced PAH [23]. 17i, an HSP110/sGC dual-target modulator, can also markedly alleviate pulmonary vascular remodeling and right ventricular hypertrophy and effectively reduce right ventricular systolic pressure in hypoxia-induced PAH rat models [90]. Given the high conservation of vascular remodeling mechanisms between PAH and systemic hypertension, these HSP110 inhibitors are promising lead compounds for hypertension treatment.

The interaction between HSP70 and HOP is a key step for the assembly of the HSP70-HSP90 molecular chaperone complex, as well as a core link in regulating cellular protein folding. Compared with directly targeting the ATP-binding domain of HSPs, blocking the HSP70-HOP interaction confers higher specificity and can markedly reduce off-target risks. At present, researchers have successfully screened multiple small-molecule compounds capable of regulating this interaction through structure-based drug design. Compound C1 stabilizes the HSP70-HOP complex and inhibits its protein-folding activity, whereas SY7 and SY8 act as competitive inhibitors to block the binding between the two proteins [91]. Although the efficacy of these compounds in vivo has not been evaluated in hypertension models, they provide vital lead compounds for the development of novel and highly specific HSP70 inhibitors.

Given that hypertension is a complex disease mediated by multiple factors and signaling pathways, single-target inhibitors often fail to achieve optimal antihypertensive effects. Therefore, the development of multi-target drugs has become a core research direction in the field of antihypertensive pharmacology. For example, HS56, a multi-target inhibitor that concurrently blocks Pim kinase and DAPK3, can suppress excessive vascular smooth muscle contraction by regulating the phosphorylation level of myosin light chain 20 (MLC20). It thereby reduces blood pressure in mice in a dose-dependent manner with no impact on heart rate [101]. This design strategy of multi-target synergism also provides important references for the optimization of HSP inhibitors. Specifically, concurrent targeting of HSPs and other key pathogenic pathways involved in hypertension can yield dual benefits, including blood pressure reduction and protection against target organ damage.

Despite the well-demonstrated experimental potential of small-molecule inhibitors targeting HSPs for hypertension treatment, their clinical translation still faces multiple challenges. HSPs exhibit a dual nature in their physiological functions, playing a pivotal role in the stress response of normal cells and the maintenance of protein homeostasis [102]. Nonspecific inhibition of HSPs may induce severe off-target effects, such as impairing the survival and repair functions of cardiomyocytes and increasing the risk of adverse cardiovascular events. Therefore, developing highly specific inhibitors through structural optimization remains an important objective. In addition, designing bifunctional or multifunctional inhibitors that can simultaneously regulate HSPs and other key targets based on the pathological network of hypertension may achieve synergistic blood pressure reduction and vascular protection. These approaches may provide novel breakthroughs for drug development.

### 5.3. Potential of Natural Products in Regulating HSP Expression for the Treatment of Hypertension

Natural products, by virtue of their multi-component and multi-target action, have exhibited broad application prospects in regulating HSP expression and the treatment of hypertension [103]. A large number of studies have confirmed that natural products, including active components of traditional Chinese medicine and plant-derived active substances, can exert antihypertensive effects by precisely regulating the expression levels of various HSP subtypes, ameliorating vascular remodeling and inhibiting oxidative stress.

Quercetin, the most abundant flavonoid in the human diet, is widely found in various fruits and vegetables, and its antioxidative and anti-inflammatory properties are associated with the prevention and treatment of cardiovascular diseases [104]. In vivo studies on sodium fluoride-induced hypertension have demonstrated that quercetin treatment significantly upregulates HSP70 expression in the heart and kidney, accompanied by the restoration of blood pressure, reduction in oxidative stress markers, and amelioration of histopathological damage [18]. I3C is a phytochemical extracted from cruciferous vegetables. It can lower the systolic blood pressure and the incidence of arrhythmia in SHRs and alleviate hypertension-induced cardiac remodeling. Enhanced HSP70 expression is the key mechanism underlying the protective effects of I3C, besides its anti-inflammatory and antioxidant properties [92]. Baicalin is a herb-derived flavonoid. Studies have found that baicalin exerts no significant blood pressure-lowering effect on renovascular hypertensive rats but can markedly reduce the expression of GRP78 (a member of the HSP70 family) in the myocardium, alleviate endoplasmic reticulum stress, and reduce cardiomyocyte apoptosis and fibrosis, thereby improving left ventricular remodeling [93]. Grape seed proanthocyanidins are natural polyphenols extracted from grape seeds. Although relevant studies mainly focus on PAH, they provide valuable references for research on systemic hypertension. Studies have provided evidence that grape seed proanthocyanidins can reverse pulmonary vascular remodeling and right ventricular hypertrophy in PAH rat models by downregulating HSP70 expression [105]. This effect may be associated with the inhibition of proliferation and migration in pulmonary arterial smooth muscle cells.

Existing studies have revealed a prominent paradox in the roles of the HSP70 family in hypertension and cardiovascular diseases: both upregulation and downregulation of HSP70 exert distinct cardioprotective effects. This apparent contradiction suggests that the relationship between HSP70 expression level and cardiovascular benefit is not linear but rather U-shaped or context-dependent. A unifying hypothesis is that the optimal HSP70 level varies with disease stage, cell type, and the nature of the stressor. Testing this hypothesis may require inducible, cell-type-specific HSP70 overexpression or knockdown at different time points during the progression of hypertension. Moreover, the finding that baicalin markedly ameliorates left ventricular remodeling without significant impacts on blood pressure further suggests that various members of the HSP70 family may play independent roles in blood pressure regulation and protection against target organ damage.

Further, geraniin is a compound isolated from *Geranium seemannii* Peyr, a perennial plant native to central Mexico. In vitro studies have demonstrated that geraniin can bind to the ATP-binding domain (N-terminal region) of the HSP90α protein, effectively inhibiting the ATPase activity and chaperone function of HSP90α [106]. In vivo studies have shown that geraniin exerts diuretic effects similar to those of first-line drugs for treating essential arterial hypertension; this may be related to its inhibition of the chaperone activity of HSP90, suggesting its potential role in the treatment of hypertension or edematous states [94].

Natural products can regulate the expression of HSPs through multiple mechanisms, thereby ameliorating hypertension-related damage and oxidative stress and exhibiting remarkable therapeutic potential. Different types of natural products have unique advantages in regulating HSP subtypes and their associated signaling pathways.

### 5.4. Translational, Safety, Ethical, and Regulatory Hurdles for HSP-Targeted Therapies

Despite promising preclinical evidence for targeting HSPs in hypertension, multiple translational, safety, ethical, and regulatory hurdles must be addressed before clinical implementation.

Translating HSP-targeted strategies from bench to bedside faces multiple gaps. Most preclinical evidence comes from AngII-infused or spontaneously hypertensive rodent models, which incompletely recapitulate the polygenic and environmental heterogeneity of hypertension in humans. Overcoming these translational barriers may require long-term efficacy studies in large animal models, formulation redesign for chronic oral administration, and prospective identification of responder subgroups.

The physiological pleiotropy of HSPs presents a paramount safety challenge. Although several investigational HSP90 inhibitors have demonstrated clinical efficacy, both off-target toxicities and drug-related on-target toxicities have posed formidable challenges to their clinical development. Specifically, inhibition of the HSP90α isoform induces both ocular and cardiac toxicities. Cellular experiments have confirmed that the maturation and membrane-trafficking processes of the hERG channel are specifically dependent on the HSP90α isoform. Broad-spectrum HSP90 inhibitors suppress HSP90α function and disrupt the normal expression and trafficking of hERG channels. This consequently induces cardiac adverse events such as long QT syndrome and cardiac arrhythmias, severely limiting the clinical application of this class of agents [107]. In another in vivo animal study, HSP90α was identified as a critical molecule for maintaining the normal structure and function of retinal photoreceptor cells. Inhibition of HSP90α downregulates microtubule-associated protein 1B (MAP1B) and triggers microtubule destabilization, which in turn leads to Golgi apparatus fragmentation and vesicular trafficking impairment, ultimately resulting in photoreceptor apoptosis and retinal degeneration [108]. Non-specific systemic targeting of HSPs could inadvertently impair beneficial physiological functions such as apoptosis regulation, protein quality control, and adaptive stress responses.

Therapeutic strategies targeting HSP genes hold promise for achieving durable blood pressure control, yet they raise significant ethical and regulatory concerns. For instance, similar to all cutting-edge technologies, gene editing is inherently a double-edged sword. As gene editing systems continue to be iteratively refined, increasingly simpler technical operations and a drastically lowered accessibility threshold have led to further amplification of the potential for misuse of this technology, posing formidable challenges to global biosecurity governance. Currently, no regulatory framework specifically addresses HSP-targeted therapies for hypertension. Drug regulatory agencies have approved HSP90 inhibitors only for cancer indications, where the risk–benefit balance favors accepting higher toxicity. In hypertension, more stringent long-term safety requirements apply, and dosing regimens must be optimized to avoid continuous perturbation of protein homeostasis. Natural products modulating HSP expression face distinct regulatory hurdles, including batch-to-batch variability, lack of standardized extraction and quality control, and the inherent complexity of multi-component mixtures. Although randomized controlled trials of some herbal formulations have reported acceptable safety profiles in patients with hypertension, regulatory approval as standardized therapeutics requires rigorous chemical fingerprinting and reproducible manufacturing processes.

## 6. Conclusions

HSPs exert distinct subtype-specific regulatory effects on the core pathological processes of hypertension, including endothelial dysfunction, abnormal proliferation and migration of VSMCs, oxidative stress, and inflammatory activation. Also, their abnormal expression is closely linked to hypertension progression and target organ damage. HSP90α, HSP70, and other key subtypes exhibit immense potential as novel biomarkers for the early diagnosis, dynamic monitoring, and prognostic assessment of hypertension. Further, gene editing technologies, HSP-targeted small-molecule inhibitors and natural products (especially traditional Chinese medicine active components) have demonstrated promising therapeutic effects in preclinical hypertension research, with combined intervention strategies further improving blood pressure control and protection against target organ damage.

However, the clinical translation of HSP-based hypertension diagnosis and treatment still faces multiple challenges. These include the unclear clinical threshold of the double-edged sword effect of HSPs, the lack of highly specific HSP modulators, and the need for further standardization of the HSP biomarker detection system. Future studies should focus on elucidating the cell- and stage-specific molecular regulatory networks of key HSP subtypes, developing highly specific and low-toxicity HSP-targeted drugs, and optimizing the delivery system of gene editing technologies. Large-sample, multicenter clinical trials are urgently needed to verify the diagnostic and therapeutic value of HSPs and to establish an HSP-based multidimensional evaluation system for hypertension. Through interdisciplinary collaboration and the integration of basic research and clinical practice, HSP-related research results can be translated into clinical practice, providing novel strategies for the precise prevention and treatment of hypertension and the protection of target organs from damage.

## Figures and Tables

**Table 1:** Key HSP Isoforms and Their Associations with Core Pathological Processes in Hypertension.

HSP Isoform	Regulated Pathological Process	Molecular Mechanism	Title	Year	Author	Ref.
HSP90α	Endothelial dysfunction	Stabilizes eNOS and Enhances NO bioavailability	Serum Heat Shock Protein 90 Alpha: A New Marker of Hypertension-Induced Endothelial Injury?	2016	Skorzynska-Dziduszko et al.	[11]
	VSMC proliferation/migration	Abnormally highly expressed in atherosclerotic lesions; inhibition induces G1-phase cell cycle arrest in VSMCs	The Role of Heat Shock Protein 90 in Migration and Proliferation of Vascular Smooth Muscle Cells in the Development of Atherosclerosis.	2014	Kim et al.	[12]
	Oxidative stress	Facilitates the assembly of the Akt-eNOS complex and mediates NO release	Dominant-Negative HSP90 Reduces VEGF-Stimulated Nitric Oxide Release and Migration in Endothelial Cells.	2008	Miao et al.	[13]
		Secreted by VSMCs under oxidative stress to activate ERK1/2 pathway	Purification and Identification of Secreted Oxidative Stress-Induced Factors from Vascular Smooth Muscle Cells.	2000	Liao et al.	[14]
	Inflammation	Downregulates the protein levels of IKK-α and IKK-β and inhibits NF-κB activation	Heat Shock Protein 90 Inhibition by 17-DMAG Attenuates Abdominal Aortic Aneurysm Formation in Mice	2015	Qi et al.	[15]
HSP70	Endothelial dysfunction	Reduce damage to the homeostasis of endothelial cells	Shear Stress-Exposed Pulmonary Artery Endothelial Cells Fail to Upregulate HSP70 in Chronic Thromboembolic Pulmonary Hypertension.	2020	Salibe-Filho et al.	[16]
	VSMC hypertrophy	Promotes MKP-1 accumulation to inactivate ERK1/2	Inhibitory Effect of HSP70 on AngII-Induced Vascular Smooth Muscle Cell Hypertrophy	2006	Zheng et al.	[9]
	Oxidative stress	Synergizes with CHIP to degrade Nox4;	Heat Shock Protein 70 and CHIP Promote Nox4 Ubiquitination and Degradation within the Losartan Antioxidative Effect in Proximal Tubule Cells.	2015	Gil Lorenzo et al.	[17]
		Upregulated by quercetin to suppress NaF-induced oxidative stress	Quercetin Attenuates Hypertension Induced by Sodium Fluoride via Reduction in Oxidative Stress and Modulation of HSP 70/ERK/PPARγ Signaling Pathways.	2018	Oyagbemi et al.	[18]
		Induced by repetitive hyperthermia to inhibit high-salt-induced cardiac oxidative stress	Repetitive Hyperthermia Attenuates Progression of Left Ventricular Hypertrophy and Increases Telomerase Activity in Hypertensive Rats.	2012	Oyama et al.	[19]
	Inflammation	Acts as autoantigen to activate CD4+ T cells	Immune Reactivity to Heat Shock Protein 70 Expressed in the Kidney Is Cause of Salt-Sensitive Hypertension.	2013	Pons et al.	[10]
HSPA12B (HSP70 family)	Endothelial dysfunction	Activates VEGF signaling pathway to promote endothelial cell proliferation and migration	Alpha-Lipoic Acid Inhibits Proliferation and Migration of Human Vascular Endothelial Cells through Downregulating HSPA12B/VEGF Signaling Axis.	2020	Ni et al.	[20]
HSPA5 (HSP70 family)	Endothelial dysfunction	Stabilizes PIEZO1 to activate Akt-eNOS signaling axis	ALDH2 Rs671 Variant Lowers Blood Pressure Via the Endothelial HSPA5-PIEZO1-eNOS Pathway.	2026	Ye et al.	[5]
HSPA1L (HSP70 family)	Oxidative stress	Interacts with VEGFR3 to enhance mitophagy and reduce ROS	VEGFR3 Mitigates Hypertensive Nephropathy by Enhancing Mitophagy via Regulating Crotonylation of HSPA1L.	2025	Wu et al.	[7]
HSP72 (HSP70 family)	Oxidative stress	Induced by lipid peroxidation in the frontal cortex and hippocampus of SHR as a biomarker of cellular injury	Strain-Dependent Effects of Long-Term Treatment with Melatonin on Kainic Acid-Induced Status Epilepticus, Oxidative Stress and the Expression of Heat Shock Proteins	2013	Atanasova et al.	[21]
HSP27	VSMC proliferation/migration	Regulates actin cytoskeleton rearrangement	Role of Heat Shock Protein 27 Phosphorylation in Migration of Vascular Smooth Muscle Cells.	2009	Chen et al.	[22]
	Inflammation	Inhibits NF-κB signaling pathway	Heat Shock Treatment Protects against AngII-Induced Hypertension and Inflammation in Aorta.	2004	Chen et al.	[8]
HSP110	Endothelial dysfunction	Interacts with STAT3 to upregulate pSTAT3 and c-Myc, inducing abnormal proliferation and migration of endothelial cells	Inhibition of HSP110-STAT3 Interaction in Endothelial Cells Alleviates Vascular Remodeling in Hypoxic Pulmonary Arterial Hypertension Model.	2023	Zhao et al.	[23]
HSF1 (HSP upstream transcription factor)	VSMC proliferation/migration	Activated by AngII via AT1R-ERK1/2 pathway; transcriptionally upregulates FAM3A to promote ATP production and release	VSMC-Specific Deletion of FAM3A Attenuated AngII-Promoted Hypertension and Cardiovascular Hypertrophy.	2020	Xiang et al.	[6]
	Oxidative stress	Nuclear translocation activates UPRmt pathway; upregulates ATF5, HSP60 and SOD2	Metformin Induction of Heat Shock Factor 1 Activation and the Mitochondrial Unfolded Protein Response Alleviate Cardiac Remodeling in Spontaneously Hypertensive Rats	2024	Xu et al.	[24]
	Inflammation	Inhibits NF-κB signaling pathway	Small Interfering RNA Knocks down Heat Shock Factor-1 (HSF-1) and Exacerbates pro-Inflammatory Activation of NF-κB and AP-1 in Vascular Smooth Muscle Cells.	2006	Chen et al.	[25]

HSP, Heat Shock Protein; HSP90α, Heat Shock Protein 90 Alpha; eNOS, endothelial Nitric Oxide Synthase; NO, Nitric Oxide; VSMC, Vascular Smooth Muscle Cell; ERK1/2, Extracellular Signal-Regulated Kinase 1/2; Akt, Protein Kinase B; IKK-α, IκB Kinase α; IKK-β, IκB Kinase β; 17-DMAG, 17-Dimethylaminoethylamino-17-Demethoxygeldanamycin; NF-κB, Nuclear Factor Kappa-B; HSP70, Heat Shock Protein 70; CHIP, Carboxy Terminus of HSP70-Interacting Protein; Nox4, NADPH Oxidase 4; NaF, Sodium Fluoride; PPARγ, Peroxisome Proliferator-Activated Receptor γ; HSPA12B, Heat Shock 70 kDa Protein 12B; VEGF, Vascular Endothelial Growth Factor; HSPA5, Heat Shock 70 kDa Protein 5; PIEZO1, Piezo Type Mechanosensitive Ion Channel Component 1; HSPA1L, Heat Shock 70 kDa Protein 1L; VEGFR3, Vascular Endothelial Growth Factor Receptor 3; ROS, Reactive Oxygen Species; HSP72, Heat Shock Protein 72; SHR, Spontaneously Hypertensive Rat; HSP27, Heat Shock Protein 27; HSP110, Heat Shock Protein 110; STAT3, Signal Transducer and Activator of Transcription 3; c-Myc, c-Myelocytomatosis Oncogene Protein; HSF1, Heat Shock Factor 1; AT1R, Angiotensin II Type 1 Receptor; FAM3A, Family With Sequence Similarity 3 Member A; UPRmt, Mitochondrial Unfolded Protein Response; ATF5, Activating Transcription Factor 5; HSP60, Heat Shock Protein 60; SOD2, Superoxide Dismutase 2; AP-1, Activator Protein 1.

**Table 2:** Clinical Biomarker Potential of HSP Isoforms in Hypertension.

HSP Isoform	Clinical Application Scenario	Expression Change	Title	Year	Author	Ref.
HSP70	Early diagnosis	Elevated HSP70 expression correlates positively with inflammatory markers.	Expression of Heat Shock Protein 70 Gene and Its Correlation with Inflammatory Markers in Essential Hypertension.	2016	Srivastava et al.	[68]
		Elevated plasma HSP70 mRNA levels in gestational hypertension	Circulating Heat Shock Protein mRNA Profile in Gestational Hypertension, Pre-Eclampsia & Foetal Growth Restriction.	2016	Hromadnikova et al.	[69]
	Disease monitoring	Elevated in rat aorta after acute hypertension	Acute Hypertension Induces Heat-Shock Protein 70 Gene Expression in Rat Aorta	1995	Xu et al.	[70]
		Elevation accelerates cardiac remodeling	Asb10 Accelerates Pathological Cardiac Remodeling by Stabilizing HSP70.	2025	Lin et al.	[71]
	Prognostic assessment	Elevated serum HSP70 increases the risk of atrial fibrillation in hypertensive patients.	Heat Shock Protein 70 Is Associated With Cardioversion Outcome and Recurrence of Symptomatic Recent Onset Atrial Fibrillation in Hypertensive Patients.	2021	Rigopoulos et al.	[72]
		Baseline levels predict atherosclerosis progression in hypertensive patients	Serum Heat Shock Protein 70 Levels Predict the Development of Atherosclerosis in Subjects with Established Hypertension.	2003	Pockley et al.	[73]
HSP90α	Early diagnosis	Elevated in serum as a marker of endothelial injury	Serum Heat Shock Protein 90 Alpha: A New Marker of Hypertension-Induced Endothelial Injury?	2016	Skórzyńska-Dziduszko et al.	[11]
	Prognostic assessment	Elevated in serum; positively correlated with left ventricular hypertrophy	Cardiac Secreted HSP90α Exacerbates Pressure Overload Myocardial Hypertrophy and Heart Failure.	2025	Pan et al.	[74]
HSP60	Early diagnosis	Elevated in borderline hypertension	Circulating Heat Shock Protein 60 Is Associated with Early Cardiovascular Disease.	2000	Pockley et al.	[75]
HSP27	Disease monitoring	Bidirectional change with age	Small Heat Shock Proteins HSP10 and HSP27 in the Left Ventricular Myocardium in Rats with Arterial Hypertension and Insulin-Dependent Diabetes Mellitus.	2021	Sklifasovskaya et al.	[76]
HSP72 (HSP70 family)	Disease monitoring	Inducibility decreases with aging in SHR myocardium	Hypertension, Aging, and Myocardial Synthesis of Heat-Shock Protein 72.	1994	Bongrazio et al.	[77]
eHSP72	Prognostic assessment	Plasma extracellular eHSP72 levels correlate with antioxidant enzyme activity in hypertensive patients	Detectable Levels of eHSP72 in Plasma Are Associated with Physical Activity and Antioxidant Enzyme Activity Levels in Hypertensive Subjects.	2018	Ten Caten Martins et al.	[78]

HSP, Heat Shock Protein; HSP70, Heat Shock Protein 70; HSP90α, Heat Shock Protein 90 Alpha; HSP60, Heat Shock Protein 60; HSP27, Heat Shock Protein 27; HSP72, Heat Shock Protein 72; eHSP72, Extracellular Heat Shock Protein 72.

**Table 3:** Targeted Intervention Strategies for HSPs in Hypertension.

Intervention Type	Target/Regulatory Object	Specific Intervention	Core Mechanism	Title	Year	Author	Ref.
Gene editing	HSP90α	CRISPR/Cas9-mediated knockout	Precise regulation of HSP gene expression	Remotely Sequential Activation of Biofunctional MXenes for Spatiotemporally Controlled Photothermal Cancer Therapy Integrated with Multimodal Imaging.	2025	Jia et al.	[87]
Small-molecule inhibitors	HSP90	17-AAG	Inhibits HSP90 chaperone function	Myocyte-Derived HSP90 Modulates Collagen Upregulation via Biphasic Activation of STAT-3 in Fibroblasts during Cardiac Hypertrophy.	2017	Datta et al.	[88]
		17-DMAG	Blocks mitochondrial fission pathways	Targeting HSP90 Attenuates AngII-Induced Adventitial Remodelling via Suppression of Mitochondrial Fission.	2020	Huang et al.	[89]
			Inhibit AngII-induced inflammatory response	Heat Shock Protein 90 Inhibition by 17-DMAG Attenuates Abdominal Aortic Aneurysm Formation in Mice.	2015	Qi et al.	[15]
	HSP110	HSP110/STAT3 inhibitors	Disrupts HSP110-STAT3 complex formation	Inhibition of HSP110-STAT3 Interaction in Endothelial Cells Alleviates Vascular Remodeling in Hypoxic Pulmonary Arterial Hypertension Model.	2023	Zhao et al.	[23]
		17i	Dual targeting of HSP110 and sGC	Discovery and Optimization of HSP110 and sGC Dual-Target Regulators for the Treatment of Pulmonary Arterial Hypertension.	2024	Hu et al.	[90]
	HSP70	C1; SY7; SY8	Stabilizes or blocks HSP70-HOP complex assembly	Designing de Novo Small Molecules That Control Heat Shock Protein 70 (HSP70) and Heat Shock Organizing Protein (HOP) within the Chaperone Protein-Folding Machinery.	2019	Zaiter et al.	[91]
Natural products	HSP70	Quercetin	Upregulates HSP70 expression	Quercetin Attenuates Hypertension Induced by Sodium Fluoride via Reduction in Oxidative Stress and Modulation of HSP 70/ERK/PPARγ Signaling Pathways	2018	Oyagbemi et al.	[18]
		Indole-3-carbinol (I3C)	Upregulates HSP70 expression	Anti-Inflammatory, Antioxidant, Antihypertensive, and Antiarrhythmic Effect of Indole-3-Carbinol, a Phytochemical Derived from Cruciferous Vegetables.	2022	Prado et al.	[92]
	GRP78 (HSP70 family)	Baicalin	Downregulates GRP78 expression Alleviates endoplasmic reticulum stress	Effects of Baicalin on Blood Pressure and Left Ventricular Remodeling in Rats with Renovascular Hypertension.	2017	Dai et al.	[93]
	HSP90α	Geraniin	Exerts diuretic effect similar to first-line antihypertensive drugs	Geraniin Is a Diuretic by Inhibiting the Na+-K+-2Cl- Cotransporter NKCC2.	2018	Moreno et al.	[94]

CRISPR/Cas9, Clustered Regularly Interspaced Short Palindromic Repeats/CRISPR-Associated Protein 9; HSP70, Heat Shock Protein 70; HSP90α, Heat Shock Protein 90 Alpha; HSP110, Heat Shock Protein 110; HOP, Heat Shock Organizing Protein; GRP78, Glucose-Regulated Protein 78; 17-AAG, 17-Allylamino-17-Demethoxygeldanamycin; 17-DMAG, 17-Dimethylaminoethylamino-17-Demethoxygeldanamycin; STAT3, Signal Transducer and Activator of Transcription 3; sGC, Soluble Guanylate Cyclase; I3C, Indole-3-Carbinol; NKCC2, Na^+^-K^+^-2Cl^−^ Cotransporter 2.

## Data Availability

No new data were created or analyzed in this study.
